# Spatial Patterns and Environmental Control of Polychaete Communities in the Southwestern Barents Sea

**DOI:** 10.3390/biology13110924

**Published:** 2024-11-13

**Authors:** Dinara R. Dikaeva, Alexander G. Dvoretsky

**Affiliations:** Murmansk Marine Biological Institute of the Russian Academy of Sciences (MMBI RAS), 183038 Murmansk, Russia

**Keywords:** seafloor, community structure, monitoring, environmental factors, Kola Transect, Arctic

## Abstract

The Barents Sea region is influenced by an increased inflow of warm Atlantic water, which impacts all components of the local ecosystem. Information on the state of benthic communities is required to predict alterations in the food web’s structure and functioning. The spatial distribution of polychaete communities was investigated in relation to environmental conditions at nine stations along the Kola Transect (70°00′–74°00′ N, 33°30′ E) in April 2019. A taxonomically diverse fauna containing 114 taxa was found, with 95 identified at the species level. The fauna was composed predominantly of boreo-Arctic species (63%), followed by boreal (22%) and Arctic species (13%). The polychaete abundance and biomass exhibited considerable variability, ranging from 910 to 3546 ind. m^−2^ and from 3.4 to 72.7 g m^−2^, with average values of 1900 ind. m^−2^ and 18.7 g m^−2^, respectively. Cluster analysis revealed three distinct polychaete communities differing in dominant species composition, abundance, and biomass. The southern region featured the most abundant community, the middle part exhibited the highest diversity, and the northern area presented the community with the highest biomass. These spatial variations in community structure corresponded closely to the distribution and properties of water masses within the study area. Multivariate analysis identified depth as the primary driver of diversity indices, with higher values observed at shallow water sites. Salinity and water temperature together explained 46% of the variation in abundance, reflecting warming effects and showing positive or negative effects, depending on the taxa. Furthermore, an increase in water temperature had a positive impact on the contribution of boreal species to the total material, while exerting a strong negative effect on the overall community biomass, underscoring the potential of polychaetes in biological indication.

## Simple Summary

The ecosystem of the Barents Sea region is facing alterations due to the increased inflow of warm Atlantic water, necessitating an understanding of the structure of seafloor communities to predict changes in the local food web. Our investigation of polychaete communities along the Kola Transect revealed a taxonomically diverse fauna, with a predominantly boreo-Arctic species composition. The study found considerable variability in polychaete abundance and biomass, with distinct community structures corresponding to the distribution and properties of water masses within the study area. Depth was identified as the primary driver of diversity indices, while salinity and water temperature collectively explained a significant portion of the variation in abundance. The increased water temperatures due to warming were unfavorable for Arctic species. The findings of this study have important implications for understanding the impact of environmental changes on benthic communities in the Barents Sea region. The identification of polychaetes as potential biological indicators highlights their importance in monitoring ecosystem changes.

## 1. Introduction

Covering about one third of the EartH′s oceanic shelves, the Arctic Ocean features areas where more than half have bathymetric depths not reaching beyond 200 m. This region displays periodic or constant ice coverage, severe thermal conditions, and significant temporal fluctuations in their light regimes, demonstrating the stark contrast between the polar night and the midnight sun [1]. The Arctic Ocean undergoes fluctuating influxes of organic matter [2]. Specifically, in high-latitude regions, the presence and survival of organisms are significantly influenced by the cyclical nature of their environmental characteristics and the resulting temporal patterns in nutrient availability [1].

The Barents Sea is a unique inflow shelf sea known for its substantial biological productivity [3]. It has an average annual primary productivity of 90 g C m^−2^ yr^−1^, as calculated by Sakshaug [4]. A distinguished oceanographic feature named the polar front partitions the sea into discrete northern and southern regions near 75–76° N, with a distinct boundary in the western sector. To the south, the waters are mostly influenced by the Atlantic water and have a high salinity level (>34.9) and mild temperature profiles (3–6 °C). The southwestern areas are free of ice. On the other hand, the northern part is dominated by polar waters, which are characterized by lower salinity (34.3–34.8) and low temperatures, resulting from the contribution of sea ice meltwater [5,6]. Spatial gradients and environmental shifts lead to significant variability in biomass distribution and productivity within the Barents Sea ecosystem.

The deposition of primary production in the benthos ranging between 48% and 96% is a defining characteristic of the Barents Sea, depending on the prevailing hydrographic and physical conditions [3]. This heterogeneity in primary production, together with the physical habitat characteristics, supports the functioning of diverse benthic communities [7]. Temporal changes in phytoplankton primary production have been linked to a cascade of effects on the food web, ultimately impacting the zooplankton population and leading to changes in the deposition of ungrazed phytoplankton in benthic ecosystems [8,9]. Augmented primary productivity may improve pelagic food web dynamics, potentially leading to increased interception of organic matter by pelagic organisms before it reaches the seabed [10]. However, nutrient limitations may hinder this process [11]. Elevated grazing levels in the pelagic zone could reduce the carbon downward flux, potentially weakening the pelagic-benthic coupling processes. Benthic organisms found in the southern Barents Sea display an elevated level of taxonomic diversity and exhibit a wide range of trophic guilds, which include filter and suspension feeders, scavengers, as well as predators [12,13]. The trophic dynamics in this community are subject to variability, potentially reflecting seasonal physiological rhythms, the diversity of dietary inputs, and differential assimilation durations of different taxonomic or functional groups [14,15].

Within the diverse ecosystem of the Arctic Ocean, Annelida, with a significant representation of approximately 500 polychaete species, holds the status of the second most species-rich and diverse taxon after Crustacea [1]. Polychaetes are numerical dominants within most Arctic shelf systems characterized by soft sediment [16]. Polychaetes are traditionally divided into two major distinct categories. The first one is the “errant” forms, which include Phyllodocida and Eunicida, while the second one is the “sedentary” forms, which include the remaining orders. Their ecological niche varies, with some being sessile (anchored to hard substrates, as seen in Serpulidae) or residing in tubes within soft sediments while others are motile. The diversity of their feeding strategies is extensive, ranging from surface and subsurface deposit feeders to suspension feeders and encompassing herbivores, carnivores, and omnivores [17,18]. As they burrow and feed, these organisms play an active role in enhancing different sedimentary processes, including nutrient recycling, sediment reworking, irrigation, oxygenation, bioturbation, and organic matter redistribution and burial. Sessile suspension feeders on hard substrates play a crucial role in transferring energy from the pelagial to the benthic zone [1,16].

Climatic fluctuations leading to warming are suggested to cause a northward shift in the biogeographic boundaries of many benthic species [19,20,21,22,23,24]. In addition, ocean acidification, invasive species, and bottom trawling may also affect biodiversity and the functioning of benthic systems, including polychaete communities [1]. Polychaetes are frequently utilized in coastal monitoring efforts, particularly in soft-bottom habitats [25,26,27]. Within benthic communities, polychaetes are highly effective indicators of environmental disturbance, as this group contains both resilient and vulnerable species distributed along a gradient from undisturbed to heavily impacted habitats [28,29]. Polychaetes represent a significant portion of the diets of numerous predatory species. For example, this group constitutes a considerable proportion of the diet of red king crabs, with a documented frequency of occurrence as high as 35–50% in the southern Barents Sea [30,31,32].

In the southern Barents Sea, the initial comprehensive study of polychaetes and other benthic taxa was conducted by Deryugin [33]. This study reported a list comprising 53 species and delineated the distribution boundaries for subarctic species. Based on data from 1921 to 1959, Nesis [34] described variations in the proportions of boreal and Arctic species but did not identify any relationships. Since the 1990s, there has been a notable intensification of research efforts in the Kola Section, including those focused on polychaetes. This has led to the acquisition of new data on the distribution patterns of benthic organisms within the area [35]. Furthermore, Dikaeva et al. [36] and Lyubina et al. [37] observed an increase in the number of boreal species, particularly in the southern region of the Kola Section. Despite the extensive study of polychaetes in the southwestern Barents Sea [36,37,38,39], there is a need for operative information on the modern status of their communities to effectively monitor both short- and long-term changes in the Barents Sea ecosystem [40] because of significant and rapid shifts in the ecosystem associated with global warming [41,42]. In this context, our study aimed to describe spatial patterns in the diversity and abundance of polychaete communities along the Kola Transect, a standard transect situated in the southwestern Barents Sea, and to evaluate the role of environmental factors in determining their structure under modern climatic conditions.

## 2. Materials and Methods

### 2.1. Sample Collection and Processing

**Figure 1:**
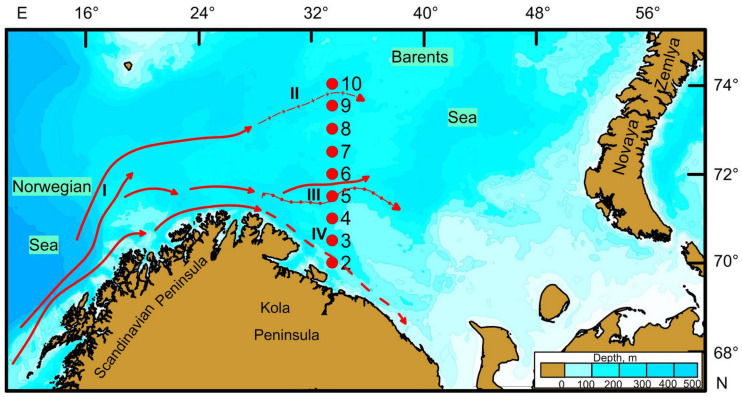
Location of standard sampling stations in the Kola Transect of the southwestern Barents Sea in April 2019. Currents: I—Norwegian Current, II—North Cape Current, III—Murmansk Current, and IV—Murmansk Coastal Current [6].

In April of 2019, a research expedition aboard the R/V Dalnie Zelentsy conducted macrozoobenthos sampling along the Kola Transect. The transect, consisting of nine stations, was sampled at roughly half-degree intervals, equating to approximately 30 nautical miles (Figure 1), and at depths ranging from 148 to 323 m. A Van Veen grab designed to collect an area of 0.1 m^−2^ was used with five replicate samples taken from each location except for station 5, where samples were taken in triplicate.

The 43 samples acquired in this study were sieved with a 0.5 mm mesh size and then preserved in 4% neutral buffered formalin. The sediment classifications and content (%) were determined by visually assessing them, in line with the criteria proposed by Istoshin [43] as described in Pavlova et al. [44]. An SBE 19plus V2 CTD Sealogger (Sea-Bird Scientific, St, Bellevue, WA, USA) was used to measure the near-bottom water temperature and salinity.

The benthic samples were washed again in the laboratory, preserved in 75% ethanol, and then separated into major benthic groups. Polychaetes were identified under a stereomicroscope to the most precise taxonomic level possible [45,46,47,48,49,50,51] and then measured and weighed with a wet weight precision of 0.0001 g. Polychaetes which produced tubes were weighed with the tubes, whereas tube-dwelling polychaetes were weighed without their tubes. The abundance (ind. m^−2^) and biomass (g m^−2^) were computed for each sample and then averaged for each station except for station 2.

Multiple diversity indices were calculated, including the species richness (SR) at each station, Shannon index (H′), Pielou evenness (J′), and total expected species number (Chao2 index).

The biogeographic classification of polychaetes was determined based on Jirkov [51].

### 2.2. Statistical Analysis

To differentiate spatial communities, cluster analysis was conducted using the Bray–Curtis similarity matrix of polychaete abundances, employing group average linkage classification. The data were fourth root-transformed prior to analysis. Hierarchical clustering-based similarities between station groups were checked via an analysis of similarities (ANOSIM). The species contribution to dissimilarities between the station groups was determined using SIMPER analysis [52]. Differences in hydrological parameters and biological data were evaluated using one-way analysis of variance (ANOVA) followed by Tukey’s multiple comparison tests. The Shapiro–Wilk test and modified Levene’s test were applied to examine the normal distribution and homoscedasticity of data. When necessary, the data were transformed to satisfy the normality and homogeneity assumptions. Differences in the diversity indices between clusters were assessed using permutation tests based on 999 random permutations.

To investigate the relationships between local environmental variables and polychaete abundances, biomasses, and diversity indices, a redundancy analysis (RDA) was conducted. This approach was preferred based on preliminary detrended correspondence analysis, which revealed that the length of the first axis was less than three standard deviation units [53]. The environmental dataset used in the analysis comprised the parameters mentioned earlier with the content of coarse sediments as a proxy for the sediment content, while four distinct datasets were utilized to quantify the response variables. Two datasets included the abundances and biomasses of all polychaete species, while the third dataset contained the diversity indices. The fourth dataset contained the contributions of Arctic and boreal species to the total number of species, abundance, and biomass. A Monte Carlo permutation test (*n* = 999) was performed to reveal the explanatory variables which best explained the polychaete abundance, biomass, and diversity data. CANOCO for Windows v. 4.5 was utilized for all ordinations [53]. Prior to analysis, the species abundance and biomass datasets were fourth root-transformed.

The mean values are presented with standard errors.

## 3. Results

### 3.1. Environmental Conditions

The sediment composition along the Kola Transect showed uniformity, with both clay and silty materials present at each sampling location with a content of 40–50%. Site 2 was characterized by an enrichment in poriferan spicules (10%), while Site 8 had a high prevalence of coarse sand, gravel, and pebbles (20%). The bathymetric measurements indicated that Site 2 was 148 m deep, while Site 10 was 323 m deep (Table 1). The water at Site 4 reached the highest recorded temperature of 4.4 °C, while Site 10 had the lowest temperature (Table 1). Site 10 also had the highest salinity, whereas Site 2 had the lowest recorded salinity levels (Table 1).

### 3.2. Polychaete Diversity, Abundance, and Biomass

Our survey identified a total of 114 polychaete taxa from 30 families (Appendix A), with 95 taxa identified at the species level. The Chao2 index for the study area was calculated to be 139. The most diverse families were Maldanidae (17 taxa, 14.8%), Terebellidae (14 taxa, 12.2%), and Ampharetidae (10 taxa, 8.7%). Among the 95 identified species, 51 were rare, occurring at only one or two stations, while 13 species were found across all stations. Regarding biogeographic affinity, the polychaetes found in the southwestern Barents Sea exhibited a prevalence of Boreo-Arctic species of 63%, while Arctic and boreal species accounted for 13% and 22%, respectively. Cosmopolitan polychaetes constituted 2% of the identified species. The diversity metrics indicated that the highest SR was observed at station 2, consisting of 64 species, while the lowest value was recorded at station 5, with 38 species (Table 1). The maximum values of H′ and J′ were noted at station 6, with the lowest levels identified at station 10 (Table 1).

The polychaete abundance varied from 910 to 3546 ind. m^−2^ at stations 5 and 2, respectively (Table 1), with an average of 1900 ± 96 ind. m^−2^. The Oweniidae family dominated the total abundance calculated for the entire study area (3147 ind. m^−2^, 18.4%), followed by Chaetopteridae (2779 ind. m^−2^, 16.3%), Maldanidae (2399 ind. m^−2^, 14.0%), Sabellidae (1157 ind. m^−2^, 6.8%), and Amphinomidae (1045 ind. m^−2^, 6.1%). The biomass values ranged from 3.4 g m^−2^ at station 4 to 72.7 g m^−2^ at station 10, averaging 18.7 ± 7.6 g m^−2^ (Table 1). The total biomass calculated for the entire study area was mainly contributed by Chaetopteridae (91.9 g m^−2^, 54.6%), Maldanidae (22.0 g m^−2^, 13.1%), Nephtyidae (13.6 g m^−2^, 8.1%), and Spionidae (12.0 g m^−2^, 7.1%).

### 3.3. Faunal Groups

**Figure 2:**
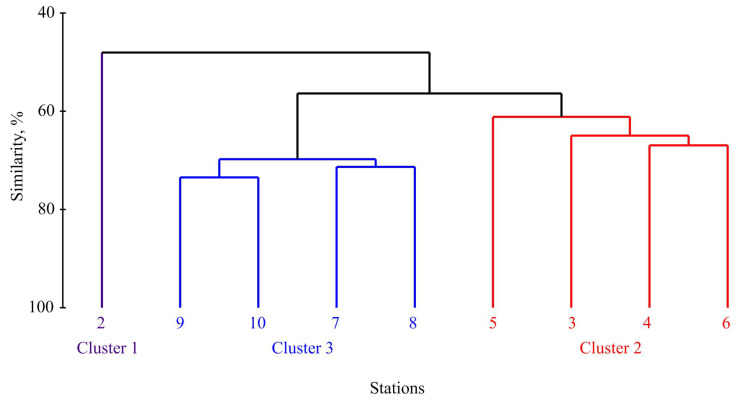
Dendrogram resulting from cluster analysis showing the similarity of polychaete fauna in the Kola Transect, performed on the Bray–Curtis similarity matrix produced from fourth root-transformed abundance data.

The cluster analysis using transformed polychaete abundance data identified three separate groups at a similarity level of 48% (Figure 2).

These groups separated station 2 (Cluster 1) from stations 3–6 (Cluster 2) and stations 7–10 (Cluster 3). Subsequent one-way ANOSIM revealed significant differences in the polychaete community composition among the delineated groups (global R = 0.882, *p* = 0.030). The numbers of unique species for Clusters 1, 2, and 3 were 13, 12, and 20, respectively. Additionally, permutation tests demonstrated significant differences in SR between Clusters 1 and 3, in H′ across all cluster pairwise comparisons, and in J′ between Clusters 2 and 3 (Table 2).

ANOVA demonstrated significant distinctions in the abundance, biomass, and oceanographic data among the clusters, with the exception of the mean biomass of Arctic species (Table 3). The observed variations were primarily due to differences between Clusters 1 and 3.

Station 2, the solitary component of Cluster 1, displayed dominance through *Chone murmanica* with 848 ind. m^−2^ (23.9%), followed by Exogoninae g. sp. (272 ind. m^−2^), *Prionospio cirrifera* (218 ind. m^−2^), and *Galathowenia fragilis* (204 ind. m^−2^). The community of Cluster 2 was primarily composed of *Maldane sarsi*, *Ophelina abranchiata*, and Lumbrineridae g. sp., with mean abundances of 229, 124, and 116 ind. m^−2^, respectively. The stations belonging to Cluster 3 featured a community of *Spiochaetopterus typicus* (average abundance of 689 ind. m^−2^), with *Galathowenia oculata* (469 ind. m^−2^) being subdominant. Furthermore, the SIMPER procedure revealed the lowest dissimilarity for the communities from Clusters 2 and 3, while the greatest dissimilarity was found for Clusters 1 and 2 (Table 4).

*Chone murmanica*, *Spiochaetopterus typicus*, Exogoninae g. sp., *Maldane* spp., *Prionospio cirrifera*, and *Galathowenia* spp. were identified as the most significant contributors to the dissimilarities between the delineated clusters within the Kola Transect (Table 4).

### 3.4. Environmental Factors Driving Polychaete Communities

**Figure 3:**
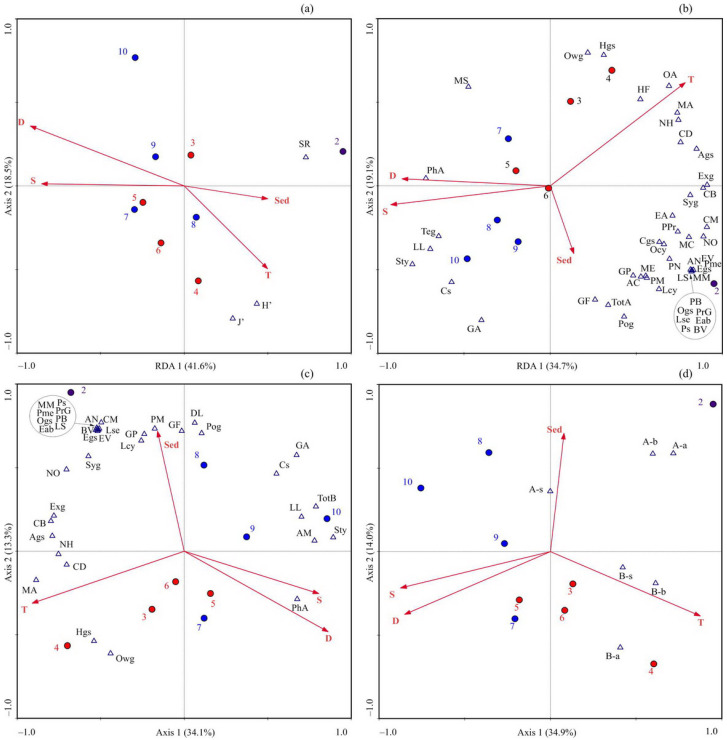
Ordination of sampling stations (represented as circles (violet—Cluster 1, red—Cluster 2, and blue—Cluster 3) by redundancy analysis with respect to polychaete diversity (**a**), abundance (**b**), biomass (**c**), and contributions of boreal and Arctic species (**d**) and their relations to environmental variables in the Kola Transect (April 2019). The proportions of the total variability explained by the first two axes are given. Environmental variables (red arrows): T—temperature, D—depth, Sed—sediments, and S—salinity. Biological variables (triangles): AC—*Amphitrite cirrata*, Ags—*Ampharetidae* g. sp., AM—*Aglaophamus malmgreni*, AN—*Aricidea nolani*, BV—*Bradabyssa villosa*, CB—*Clymenura borealis*, CM—*Chone murmanica*, Cs—*Chone* sp., DL—*Diplocirrus longisetosus*, EA—*Euchone analis*, Eab—*Ephesiella abyssorum*, Egs—Euclymeninae g. sp., EV—*Eucranta villosa*, Exg—Exogoninae g. sp., GA—*Galathowenia oculata*, GF—*Galathowenia fragilis*, GP—*Glyphanostomum pallescens*, HF—*Heteromastus filiformis*, CD—*Chone duneri*, Hgs—Hesionidae g. sp., Lcy—*Lumbriclymene cylindricaudata*, LL—*Lysippe labiata*, LS—*Lepidonotus squamatus*, Lse—*Samytha sexcirrata*, MA—*Maldane arctica*, MC—*Prionospio cirrifera*, ME—*Melinna elisabethae*, MS—*Maldane sarsi*, NH—*Nothria hyperborea*, NO—*Notoproctus oculatus*, OA—*Ophelina abranchiata*, Ogs—*Onuphidae* g. sp., Owg—*Oweniidae* g. sp., PB—*Pista bansei*, PhA—*Pholoe assimilis*, PM—*Pista maculata*, Pme—*Polycirrus medusa*, PN—*Polycirrus norvegicus*, Pog—Polynoidae g. sp., PPr—*Praxillella praetermissa*, PrG—*Proclea graffi*, Ps—*Pholoe* sp., Sty—*Spiochaetopterus typicus*, Syg—Syllidae g. sp., Teg—Terebellidae g. sp., TotA—total abundance, TotB—total biomass, H′—Shannon index, J′—Pielou evenness, SR—species richness, A-a, A-b, A-s—contributions of Arctic species to abundance, biomass, and species richness, respectively, and B-a, B-b, B-s—contributions of boreal species to abundance, biomass, and species richness, respectively.

The RDA based on polychaete diversity revealed that the first two axes accounted for a substantial proportion of the total variance in the data (RDA 1 = 41.6% and RDA 2 = 18.5%). The ordination triplot illustrated that the first axis demonstrated a strong negative correlation with depth and salinity, delineating deeper-water stations with lower SR from shallower stations with higher numbers of species. Axis 2 exhibited a negative correlation with temperature, distinguishing stations with higher H′ and J′ indices on the lower side from stations with lower values (Figure 3a).

Monte Carlo permutation tests indicated that only the depth significantly contributed to the RDA model (Table 5).

When performing RDA with polychaete abundance data, the variance explained by the first two axes totaled 53.8%. Axis 1 displayed a strong positive correlation with water temperature and a negative correlation with salinity, highlighting stations with lower salinity on the right side and stations with higher salinity on the left side (Figure 3b). Axis 2 showed a positive association with water temperature, segregating most warmer-water stations on the upper side from colder-water stations on the lower side (Figure 3b). According to the Monte Carlo permutation test, salinity and water temperature were the most significant environmental factors explaining the variance in polychaete taxa abundances (Table 5), with opposite effects depending on the species.

Furthermore, the RDA analysis conducted for the biomass data generated a model in which Axes 1 and 2 together explained 47.7% of the total variation (Figure 3c). The first axis displayed a strong negative association with water temperatures, differentiating sampling stations with lower biomass on the left side and those with higher biomass on the right side (Figure 3c). The second axis was positively correlated with the sediment content, segregating Stations 2 and 8, with a high presence of coarse material, from most other stations. The permutation test indicated water temperature as the single factor significantly affecting the polychaete biomass (Table 5).

Regarding the dataset of Arctic and boreal species’ contributions to the total SR, abundance, and biomass, the RDA revealed that the first and second canonical axes explained 49.0% of the overall variance. RDA 1 exhibited a positive correlation with water temperature and distinguished the stations of Cluster 2, which had higher contributions of boreal species, from the others (Figure 3d). RDA 2 was positively correlated with the sediment content and with contributions of Arctic species to the total material. A further test revealed that the water temperature significantly affected the biological variables (Table 5).

## 4. Discussion

### 4.1. Environmental Conditions

The environmental conditions within the study area are primarily influenced by the circulation patterns and thermohaline characteristics of water masses. The upper water layer of the Barents Sea comprises warm, salty Atlantic water in the west and cold, fresher polar water from the Arctic in the east [54]. The Norwegian Current carries warm Atlantic waters to the western part of the sea along the shelf break [55]. The largest branch of the Norwegian Current, known as the North Cape Current, enters the southwestern Barents Sea [5]. The southern branch of the North Cape Current flows eastward along the coast of northern Norway parallel to the Norwegian Coastal Current. To the northeast of the Varanger Peninsula, this southern branch moves further offshore and is renamed the Murmansk Current [6]. Another significant source of warmer water in the southwestern Barents Sea is the Norwegian Coastal Current. Within the inshore zone of the Kola Peninsula, it continues as the Murmansk Coastal Current, flowing along the peninsula to the White Sea. These coastal currents transport warm waters with relatively lower salinity from the Baltic Sea and are further augmented by freshwater from run-offs along the coast through the rivers of Norway and the Kola Peninsula [6]. In the context of the Kola Transect, Station 2 is influenced by the Murmansk Coastal Current (Figure 1), characterized by lower salinity and seasonally varying temperatures, which explains the lowest salinity and highest temperature in this region. Stations 3–7 are impacted by the main branch of the Murmansk Current, whereas Stations 8–10 lie in the zone of the colder and more saline North Cape Current [56]. Additionally, intense mixing due to strong winds and convection leads to a homogenous vertical distribution of salinity by April. Consequently, Stations 8–10 exhibit the highest salinity and lowest water temperature.

According to the findings of Karsakov et al. [56], the seasonal minimum of the bottom water temperature occurs from April (Station 2) to July (Stations 3–10), indicating that our data provide insight into the winter status of polychaete communities along the Kola Transect. Furthermore, the water temperatures for the entire month in 2019 were calculated to be 3.22 °C for Station 2, 3.73 °C for Stations 3–7, and 2.71 °C for Stations 8–10 [56]. These values were all higher than the long-term averaged values for April (2.91 °C, 3.60 °C, and 2.89 °C, respectively), suggesting that the year 2019 was warmer than usual.

### 4.2. Polychaete Diversity, Abundance, and Biomass

The Chao2 estimation indicated an expected number of species of 139, implying that our sampling campaign covered approximately 82% of the polychaete taxa within the study area during the winter season. Previous data (1995–2012) indicate that the polychaete fauna along the Kola Transect encompasses 241 taxa [36,38]. Notably, this parameter was obtained from a series of earlier benthic surveys conducted in different seasons and covering standard Stations 1 and 11, which were not examined in our study. For comparison, in the sublittoral zone of Kola Bay in 2017, the species richness was similar to that found in the Kola Transect (121) [39]. Near Novaya Zemlya, species richness was also reported to be 114 species according to data obtained in 2006, 2007, and 2019 [57], while in the northwestern part of the Barents Sea, the number of polychaete taxa was documented to range from 101 to 135 in 2005, 2015, and 2019 [58,59], which was higher than that in the northeastern Barents Sea (82) in 2019 [60], signifying differing feeding conditions in these areas [44]. The SR within the study area was determined to range from 38 to 64 taxa. Comparatively lower values of this index were found in Kola Bay (26–52 taxa), in the northwestern and northeastern parts of the Barents Sea (14–59 and 13–40 taxa, respectively), and near the Novaya Zemlya archipelago (21–40 taxa) according to surveys conducted in 2005, 2006, 2007, 2016, 2015, 2017, and 2019 [57,58,59,60,61].

The highest levels of H′ and J′, coupled with a high SR, were observed at Station 6, possibly due to the interaction between Murmansk coastal water and Atlantic water. This indicates that our study area is characterized by diverse conditions and encompasses a variety of habitats and being affected by different water masses, leading to distinct gradients in environmental conditions [56]. These conditions are favorable for benthic communities in terms of biodiversity and complex faunal structure [21]. Moreover, these biodiversity indices demonstrate fluctuations inter-annually, correlating with climate shifts which facilitate the expansion of boreal species and potentially impede the propagation of Arctic species [62].

The polychaete communities of the Kola Transect predominantly consisted of boreo-Arctic (BA) followed by boreal (Bo) and Arctic (Ar) species, with a composition of 63:22:13% (BA:Bo:Ar). This contrasts with Kola Bay, where the proportion of Arctic species was diminished (59:21:6%), and near West Spitsbergen, where a lower ratio of boreal species was observed (69:18:13–77:17:6%) [39,58,59]. The observed variances in the spatial distribution of boreal and Arctic species suggest the impact of the specific thermal properties of the water bodies, aligning with the findings for other benthic groups [62].

In general, the abundance and biomass values were similar to those in previous reports on the polychaete fauna in the Kola Transect during 2000–2015 [36,38]. In the adjacent area of Kola Bay, similar biomasses were reported but with much higher abundances of polychaete worms [39]. The mean levels in the Kola Transect were lower than those in the northwestern Barents Sea (3660 ind. m^−2^ and 50.56 g m^−2^) [58]. Additionally, higher biomass calculations (up to 190 g m^−2^) were evident in the waters off of Novaya Zemlya, while an area of the Makarov Strait with poor trophic conditions was found to have lower abundance and biomass values (up to 2550 ind m^−2^ and 46 g m^−2^, respectively) [44]. These spatial variations are mainly attributable to trophic conditions associated with primary productivity, as this factor is known to be the main driver of the survival and growth performance of benthic organisms in the Arctic [1].

### 4.3. Polychaete Communities

The spatial distributions of the abundance and biomass, along with the structural and distributional attributes of benthic communities, are indicative of both habitat conditions and trophic dynamics shaped by the characteristics of local water masses [7]. Our cluster analysis identified three distinct groups corresponding to different water masses. Specifically, Station 2 within Cluster 1 is under the influence of Murmansk coastal water, whereas the stations categorized within Clusters 2 and 3 are predominantly affected by the Atlantic water mass [56]. This has led to a discernible lower degree of faunal dissimilarity among the latter two clusters in contrast to other cluster pairs, as evidenced by multivariate comparisons (Table 4).

Cluster 1 consisted predominantly of the small sabelid polychaete *Chone murmanica*, displaying a high abundance but low biomass. This cluster was also characterized by a high proportion and abundance of boreal species (Table 3) and was associated with the shallowest site with the lowest salinity. Stations 3–6, located in the central part of the study area, formed Cluster 3, exhibiting the lowest values for the abundance and biomass and being dominated by the tube-dwelling polychaete *Maldane sarsi*. On the other hand, the deepest stations (Stations 7–10), belonging to Cluster 2, were found in colder-water sites with the greatest salinities (Table 3). This faunal complex was dominated by the tubicolous polychaete *Spiochaetopterus typicus* and demonstrated moderate abundances and the greatest biomass. The results of the cluster analysis align well with previous studies regarding the distribution of abundance and biomass, as well as the predominance of *Spiochaetopterus typicus* within the northernmost stations [36,38]. However, differences were observed in the dominating species within the southern complex and the boundary between the neighboring faunal groups in Clusters 1 and 3, which were attributed to inter-annual fluctuations in the thermohaline characteristics of the water masses in the zone where the Murmansk Current and the North Cape Current interact (Station 7) [36]. Similar patterns have been documented for the benthic fauna of the Barents Sea by other researchers [63], confirming the influence of primary productivity, ice cover, and water mass distributions on the benthic communities in Arctic shelves [7,64].

The structure of polychaete communities in the Kola Transect, characterized by the dominance of tubicolous polychaetes such as *Spiochaetopterus typicus*, *Galathowenia oculata*, and *Maldane sarsi*, as well as carnivorous polychaetes from the family Lumbrineridae in terms of abundance or biomass, mirrors similar patterns observed in other regions of the Barents Sea, including the central, northern, and eastern Barents Sea [57,60,63,65]. These species, which typically dominate the entire benthic community, play a pivotal role in the functioning of the Barents Sea food web [44].

Our investigation revealed no significant differences in the biomass of Arctic species among the clusters (Table 3), a finding consistent with the research of Norwegian authors comparing benthic macrofaunae between the northern and southern areas of the Barents Sea [63]. The southernmost station of the Kola Transect exhibited the highest contribution of boreal species to the total material, which corresponds with previous studies on polychaetes in this region and is anticipated due to the higher water temperatures in the southern sector of the sea, a favorable environment for warm-water species [7].

### 4.4. Environmental Control

Our RDA model demonstrated that variations in depth accounted for 38% of the total variation in diversity indices (Table 5), revealing a negative association between depth and SR. Similar bathymetric variations in benthic diversity indices have been documented for the northern Barents Sea [66,67]. A decrease in SR and other diversity indices with increasing depth has also been recorded along the polar front transect [65]. Depth serves as a proxy for covariant environmental factors, including sediment characteristics and the availability and quality of food resources [68]. Given the negligible variation in sediment composition across the study area, it can be hypothesized that the observed differences in biodiversity associated with depth primarily reflect alterations in trophic conditions.

Previous studies in the Kola Section have indicated an increase in the abundance and biomass of zooplankton, actively utilizing primary production from the south to the north [69]. This observation suggests lower nutrient concentrations and reduced food availability at deeper water locations, offering an explanation for the lower diversity observed in these areas. Additionally, the potential impact of the predatory red king crab should be considered. This species tends to be more abundant at shallower sites [69] and has been reported to have a negative impact on polychaetes due to grazing [30,31,32,70]. Trawl surveys have corroborated the highest abundance of red king crabs being at Station 2 [71] along with a high total abundance of 44 million red commercial male red king crabs in 2019 [72], further highlighting their potential influence on the benthic community and overall ecosystem dynamics.

According to the RDA results, salinity accounted for 31% of the total variation in the benthic community abundance (Table 5). It was observed that salinity exhibited a positive association with the abundance of certain species, including *Spiochaetopterus typicus*. Although this species has a wide tolerance to environmental conditions, it prefers more stable cold-water environments and is more abundant at offshore sites than in the coastal zone [59,73,74]. Conversely, many small, short-lived polychaetes which exhibit high abundance are more tolerant to lower salinities, enabling them to successfully occupy habitats with unstable conditions and reach high abundances, and such species demonstrated a negative correlation with salinity. Meanwhile, water temperature accounted for an additional 16% of the variability in polychaete abundance, with strong positive correlations with boreal species like *Heteromastus filiformis* and *Clymenura borealis* and a negative correlation with the Arctic species *Lysippe labiata*. Further analysis revealed that water temperature was a significant predictor in the separate RDA for Arctic versus boreal contributions, explaining 30% of the variation in SR, abundance, and biomass among boreal species, as well as the SR of Arctic polychaetes (Table 5). This relationship highlights the thermal preferences of these taxa and is consistent with trends observed in other benthic organism taxa [62,75]. Given that most polychaetes have boreo-Arctic origins, their population dynamics often reflect stability and productivity within cold-water environments with minimal temperature fluctuations. Water temperature accounted for 30% of the variability in community biomass, exhibiting a significant negative effect. This pattern is consistent with the results from other benthic groups, including bryozoans, mollusks, as well as benthic communities in general [44,62,66,70,74], and can also be explained by the local fauna’s preference for colder temperatures.

Salinity and temperature are paramount characteristics of water masses, especially salinity, which is frequently employed to distinguish different types of water masses [6,56]. The close associations observed between polychaete species and salinity and water temperature present opportunities for their potential use as biological indicators to monitor the process of Atlantification in the Barents Sea.

## 5. Conclusions

Understanding benthic community structure is critical for elucidating the consequences of ongoing climate change. Our study focused on Polychaeta, as this group plays a pivotal role in the soft-bottom benthic communities of the Barents Sea. Sampling along a standard transect situated in the southwestern part of the sea enabled us to delineate the current status of local polychaete communities and identify the primary factors driving spatial variations in diversity indices, abundance, and biomass. The polychaete fauna exhibited considerable diversity, comprising 114 taxa, consistent with other areas of the Barents Sea. However, the alpha diversity demonstrated higher values along the Kola Transect. Abundance and biomass calculations were either lower or comparable to those of other regions of the Barents Sea, except for the northern polygons with poor trophic conditions. Along the latitudinal axis, we observed three polychaete communities dominated by different taxa, displaying distinct abundance and biomass patterns and tending to decrease in abundance but increase in biomass values from south to north. These community structures were consistent with previous studies in the area, with some variations noted in the dominant taxa in the southern part and the location of cluster group boundaries. The spatial distribution of polychaete communities was associated with the primary currents and water masses, with the southern complex influenced by the Murmansk Coastal Current (Murmansk water mass), while the other groups were affected by the Murmansk Current and North Cape Currents (Atlantic water mass). Multivariate analysis identified the key environmental predictors of biological data variability: depth for diversity, salinity and temperature for abundance, and water temperature for biomass. These influences on polychaete communities mirrored those observed in other benthic groups and the Barents Sea benthos as a whole. The close associations found between water temperature and the abundance and biomass of taxa with different origins and habitat preferences may have significant implications for biological indication and the ongoing monitoring of benthic communities in the Arctic.

## Figures and Tables

**Table 1:** Near-bottom environmental conditions and polychaete abundance and biomass (mean values with standard errors) at sampling stations in the Kola Transect of the southwestern Barents Sea in April 2019.

Station	D	T	S	SR	H′	J′	Ab	Bi
2	148 ± 0.7	3.6 ± 0	34.456 ± 0	64 ± 2	3.16 ± 0.09	0.76 ± 0.05	3546 ± 486	8.71 ± 1.4
3	248 ± 0.4	3.7 ± 0	34.639 ± 0	55 ± 2	2.68 ± 0.17	0.67 ± 0.05	1650 ± 122	6.55 ± 1.13
4	214 ± 0.2	4.4 ± 0	34.685 ± 0	45 ± 1	2.85 ± 0.05	0.75 ± 0.04	1342 ± 193	3.4 ± 0.32
5	279 ± 0	2.4 ± 0	34.947 ± 0	38 ± 4	2.68 ± 0.33	0.74 ± 0.03	913 ± 197	4.12 ± 1.18
6	260 ± 0.4	2.6 ± 0	34.95 ± 0	56 ± 3	3.27 ± 0.25	0.81 ± 0.09	1488 ± 236	6.76 ± 2.14
7	280 ± 0.7	2.6 ± 0	34.934 ± 0	45 ± 1	3.02 ± 0.14	0.79 ± 0.02	1348 ± 192	17.74 ± 5.29
8	215 ± 0.5	1.5 ± 0	34.968 ± 0	50 ± 2	2.85 ± 0.14	0.73 ± 0.03	1492 ± 354	10.29 ± 2.39
9	287 ± 0.2	1.8 ± 0	34.968 ± 0	45 ± 2	2.1 ± 0.08	0.55 ± 0.17	2172 ± 309	38.24 ± 9.82
10	323 ± 1.9	1.1 ± 0	34.969 ± 0	48 ± 1	1.93 ± 0.09	0.5 ± 0.01	3144 ± 298	72.72 ± 12.62

Note: D—depth, T—temperature, S—salinity. SR—species richness, H′—Shannon index, J′—Pielou evenness, Ab—abundance (ind m^−2^), and Bi—biomass (g m^−2^).

**Table 2:** Diversity indices for the three polychaete communities in the Kola Transect in April 2019 and their pairwise comparisons.

Index	Cluster 1	Cluster 3	*p*	Cluster 1	Cluster 2	*p*	Cluster 2	Cluster 3	*p*
SR	64	83	0.018	64	73	0.123	83	73	0.2769
H′	3.156	3.292	0.003	3.156	2.521	0.0001	3.292	2.521	0.0001
J′	0.3669	0.324	0.597	0.3669	0.1705	0.0001	0.324	0.1705	0.0001

Note: SR—species richness, H′—Shannon index, J′—Pielou evenness, and *p*—probability level for permutation tests.

**Table 3:** Polychaete abundance (ind. m^−2^), biomass (g m^−2^), and environmental variables within each group, delineated via cluster analysis in the Kola Transect (Barents Sea) in April 2019, and comparisons among groups.

Variable	Cluster 1		Cluster 2		Cluster 3		ANOVA	
	Range	Mean ± SE	Range	Mean ± SE	Range	Mean ± SE	F	*p*
TotA	2240–5210	3546 ± 486	1348–3144	2039 ± 410	910–1650	1348 ± 158	9.97	0.004
Abor	310–720	470 ± 89	130–404	222 ± 38	138–292	251 ± 60	4.16	0.048
Aarct	350–1630	882 ± 233	36–86	62 ± 11	20–64	47 ± 10	34.02	<0.001
TotB	6.3–12.9	8.7 ± 1.4	10.3–72.7	37.7 ± 14.0	3.4–6.8	5.2 ± 0.8	8.28	0.008
Bbor	0.7–5.4	2.2 ± 0.8	0.3–0.9	0.6 ± 0.1	0.8–1.8	1.2 ± 0.2	6.38	0.041
Barc	0.1–1.7	1.0 ± 0.4	0.1–0.9	0.4 ± 0.2	0.1–0.4	0.2 ± 0.1	2.09	0.175
D	146–150	147.8 ± 0.7	213–323	276 ± 9	214–279	247 ± 5	34.21	<0.001
T	3.6–3.6	3.6 ± 0.0	1.1–2.6	1.8 ± 0.1	2.4–4.4	3.4 ± 0.2	27.75	<0.001
S	34.456–34.456	34.456 ± 0	34.934–34.969	34.96 ± 0.003	34.639–34.95	34.79 ± 0.03	26.08	<0.001

Note: TotA—total abundance, Abor—abundance of boreal species, Aarct—abundance of Arctic species, TotB—total biomass, Bbor—biomass of boreal species, Barct—biomass of Arctic species, T—temperature, S—salinity, D—depth, F—F ratio, and *p*—probability level. As Cluster 1 was composed of 1 station, the sample replicates were used as variables for the ANOVA.

**Table 4:** Results of SIMPER analysis, showing the contribution of polychaete taxa to the total dissimilarity between the groups delineated with cluster analysis in the Kola Transect.

Taxa	Av. Diss.	Diss/SD	Contrib%	Cum%
	Cluster 1 vs. Cluster 2, dissimilarity 79.93%			
*Chone murmanica*	15.1	7.98	18.89	18.89
*Spiochaetopterus typicus*	11.47	1.37	14.35	33.24
Exogoninae g. sp.	4.91	7.61	6.15	39.38
*Galathowenia oculata*	4.68	1.12	5.86	45.24
*Prionospio cirrifera*	3.95	6.91	4.94	50.18
*Galathowenia fragilis*	3.34	8.17	4.18	54.36
*Notoproctus oculatus*	3.05	7.26	3.82	58.18
*Maldane arctica*	2.86	8.71	3.58	61.77
Cirratulidae g. sp.	1.68	4.35	2.1	63.87
*Pista bansei*	1.67	7.26	2.09	65.96
*Ophelina abranchiata*	1.63	6.16	2.04	68.01
Syllidae g. sp.	1.46	6.8	1.82	69.83
*Leitoscoloplos acutus*	1.39	1.6	1.74	71.57
*Maldane sarsi*	1.36	0.85	1.71	73.28
*Paramphinome jeffreysii*	1.3	1.3	1.63	74.91
	Cluster 1 vs. Cluster 3, dissimilarity 74.61%			
*Chone murmanica*	16.81	12.41	22.53	22.53
Exogoninae g. sp.	5.44	12.45	7.3	29.83
*Maldane sarsi*	4.64	1.01	6.22	36.05
*Prionospio cirrifera*	4.4	12.52	5.9	41.95
*Galathowenia fragilis*	3.62	2.9	4.85	46.8
*Notoproctus oculatus*	3.4	13.32	4.56	51.36
*Galathowenia oculata*	3.1	2.56	4.15	55.51
*Maldane arctica*	2.69	3.99	3.61	59.12
*Pista bansei*	1.89	14.89	2.53	61.65
Cirratulidae g. sp.	1.63	6.14	2.18	63.83
Lumbrineridae g. sp.	1.62	2.59	2.17	66
Syllidae g. sp.	1.59	5.08	2.13	68.13
*Glyphanostomum pallescens*	1.48	11.54	1.98	70.11
*Praxillella praetermissa*	1.41	18.01	1.89	72
*Ophelina abranchiata*	1.36	1.64	1.82	73.81
	Cluster 2 vs. Cluster 3, dissimilarity 67.86%			
*Spiochaetopterus typicus*	18.25	1.64	26.89	26.89
*Galathowenia oculata*	12.16	2.46	17.92	44.81
*Maldane sarsi*	5.9	1.1	8.69	53.5
*Maldane arctica*	3.42	0.83	5.05	58.55
*Ophelina abranchiata*	2.71	1.2	4	62.54
*Paramphinome jeffreysii*	2.58	1.25	3.81	66.35
*Leitoscoloplos acutus*	1.8	1.13	2.65	69
Lumbrineridae g. sp.	1.53	1.46	2.25	71.25
*Myriochele heeri*	1.52	1.38	2.24	73.48
*Heteromastus filiformis*	1.34	1.35	1.98	75.46
*Galathowenia fragilis*	1.18	1.13	1.73	77.19
*Terebellides stroemi*	1.06	0.9	1.56	78.75
*Spiophanes kroeyeri*	1.02	1.08	1.5	80.25
*Chirimia biceps biceps*	0.68	1.82	1	81.26
Oweniidae g. sp.	0.57	1.15	0.85	82.1

Note: Av. Diss.—mean dissimilarity (%), Diss/SD—standard deviation, Contrib%—contribution to dissimilarity (%), Cum%—cumulative contribution (%).

**Table 5:** List of environmental variables which contributed to the RDA models based on the polychaete diversity, abundance, biomass, and contribution data in the Kola Section.

Variable	Diversity			Abundance			Biomass			Contribution		
	EV	F	*p*	EV	F	*p*	EV	F	*p*	EV	F	*p*
D	38	4.23	0.042	9	1.03	0.476	16	1.78	0.073	14	1.55	0.190
S	5	0.56	0.595	31	3.13	0.001	8	0.95	0.522	7	0.72	0.529
T	16	1.98	0.186	16	1.89	0.033	30	3.02	0.001	30	2.94	0.023
Sed	1	0.1	0.892	12	1.38	0.308	10	1.11	0.384	10	1.06	0.389

Note: T—temperature, S—salinity, D—depth, Sed—content of coarse sediments, EV—explained variation (%), F—pseudo-F ratio, and *p*—probability level.

## Data Availability

The data presented in this study are available on request from the corresponding author.

## Appendix A

**Table A1:** List of polychaete taxa found at stations within the Kola Transect in April 2019. “+”—presence, “−”—absence.

Taxa	Station								
	2	3	4	5	6	7	8	9	10
*Aglaophamus malmgreni* (Theel, 1879)	−	+	−	−	−	+	+	+	+
*Amage auricula* Malmgren, 1866	+	−	−	+	−	−	−	−	−
*Ampharete finmarchica* (M. Sars, 1865)	−	−	−	−	−	−	−	+	−
Ampharetidae g. sp.	+	+	+	+	+	−	−	−	−
*Amphicteis gunneri* (M. Sars, 1835)	+	+	−	−	−	−	+	−	−
*Amphitrite cirrata* Müller, 1776	+	−	−	−	−	−	+	−	−
*Anobothrus gracilis* (Malmgren, 1866)	+	−	+	−	+	+	+	+	−
*Apistobranchus tullbergi* (Théel, 1879)	−	+	+	−	+	−	−	−	+
*Aricidea hartmanae* (Strelzov, 1968)	+	+	+	+	+	+	+	+	+
*Aricidea nolani* Webster & Benedict, 1887	+	−	−	−	−	−	−	−	−
*Aricidea quadrilobata* Webster & Benedict, 1887	−	+	−	−	+	+	+	−	+
*Artacama proboscidea* Malmgren, 1866	−	−	+	−	−	−	−	−	−
*Bradabyssa villosa* (Rathke, 1843)	+	−	−	−	−	−	−	−	−
*Bylgides elegans* (Théel, 1879)	−	−	+	−	−	−	−	−	−
*Capitella capitata* (Fabricius, 1780)	−	−	−	−	−	−	−	−	+
*Chirimia biceps biceps* (Sars, 1861)	+	+	+	+	+	−	+	+	−
*Chone duneri* Malmgren, 1867	+	+	+	−	+	+	−	−	−
*Chone infundibuliformis* Krøyer, 1856	−	−	−	−	+	−	+	−	−
*Chone murmanica* Lukasch, 1910	+	+	+	+	+	+	+	+	+
*Chone* sp.	−	−	−	−	−	−	+	+	+
Cirratulidae g. sp.	+	+	+	+	+	+	+	+	+
*Cirrophorus branchiatus* Ehlers, 1908	+	+	+	+	+	+	+	+	+
*Cistenides hyperborea* Malmgren, 1866	−	+	−	−	−	−	−	−	−
*Clymenura polaris* (Théel, 1879)	−	+	−	−	−	−	−	−	−
*Clymenura* sp.	+	−	−	+	−	−	−	−	−
*Clymenura borealis* (Arwidsson, 1906)	+	−	+	−	−	−	−	−	−
*Cossura longocirrata* Webster & Benedict, 1887	−	−	−	−	−	+	−	−	+
*Diplocirrus glaucus* (Malmgren, 1867)	−	−	−	+	+	−	−	−	−
*Diplocirrus longisetosus* (Marenzeller, 1890)	+	−	−	−	+	+	+	+	+
Dorvilleidae g. sp.	−	−	−	−	+	−	+	−	−
*Ephesiella abyssorum* (Hansen, 1878)	+	−	−	−	−	−	−	−	−
*Eteone agg. flava* (Fabricius, 1780)	+	+	+	+	+	+	+	−	+
*Euchone analis* (Kröyer, 1856)	+	+	+	−	+	−	+	−	+
Euclymeninae g. sp.	+	−	−	−	−	−	−	−	−
*Eucranta villosa* Malmgren, 1865	+	−	−	−	−	−	−	−	−
Exogoninae g. sp.	+	+	+	−	+	+	−	−	−
Fabricinae g. sp.	−	−	−	+	−	−	−	−	−
*Flabelligera affinis* M. Sars, 1829	−	−	+	−	−	−	−	−	−
*Galathowenia oculata* (Zachs, 1923)	+	+	−	+	+	+	+	+	+
*Galathowenia fragilis* (Nilsen & Holthe, 1985)	+	−	−	−	+	+	+	+	+
*Gattyana amondseni* (Malmgren, 1867)	−	−	+	−	−	−	−	−	−
*Gattyana cirrhosa* (Pallas, 1766)	−	+	−	−	−	−	−	−	−
*Glycera lapidum* Quatrefages, 1866	+	+	−	+	−	−	+	−	−
*Glyphanostomum pallescens* (Théel, 1879)	+	+	+	−	+	+	+	+	+
*Harmothoe imbricata* (Linnaeus, 1767)	−	−	−	−	−	−	+	−	−
Hesionidae g. sp.	−	+	+	−	−	−	−	−	−
*Heteromastus filiformis* (Claparède, 1864)	+	+	+	+	+	+	−	+	+
*Lanassa venusta venusta* (Malm, 1874)	−	+	−	−	+	−	−	−	−
*Laonice cirrata* (M. Sars, 1851)	−	−	−	−	−	+	−	+	+
*Laonome kroeyri* Malmgren, 1866	−	−	−	−	+	−	−	−	−
*Laphania boecki* Malmgren, 1866	−	+	+	−	+	+	+	+	−
*Leaena ebranchiata* (M. Sars, 1865)	+	−	−	−	−	+	−	+	−
*Leitoscoloplos acutus* (Verrill, 1873)	+	+	+	+	+	+	+	+	+
*Lepidonotus squamatus* (Linnaeus, 1758)	+	−	−	−	−	−	−	−	−
*Levinsenia gracilis* (Tauber, 1879)	+	+	+	+	+	+	+	+	+
*Lumbriclymene minor* Arwidsson, 1906	−	+	−	+	−	−	−	+	+
*Lumbriclymene cylindricauda* Sars, 1872	+	+	+	+	+	+	+	+	−
Lumbrinereidae g. sp.	−	+	+	+	+	+	+	+	+
*Lysippe labiata* Malmgren, 1866	−	−	−	−	+	+	+	+	+
*Maldane arctica* Detinova, 1985	+	+	+	+	+	+	+	−	−
*Maldane sarsi* Malmgren, 1865	−	+	+	+	+	+	+	+	+
*Maldanidae* g. sp.	−	+	−	−	+	+	−	−	−
*Melinna elisabethae* McIntosh, 1914	+	+	−	−	+	−	−	+	+
*Micronephthys minuta* (Théel, 1879)	+	−	−	−	−	−	−	−	−
*Myriochele heeri* Malmgren, 1867	+	+	+	+	+	+	+	+	+
*Nephtys ciliata* (Müller, 1776)	+	+	+	−	+	+	+	+	+
*Nephtys paradoxa* Malm, 1874	−	−	−	−	−	−	+	+	−
*Nicomache lumbricalis* (Fabricius, 1780)	−	+	−	−	+	−	−	+	−
*Nothria hyperborea* (Hansen, 1878)	+	+	+	+	+	−	−	−	−
*Notomastus latericeus* Sars, 1851	−	−	+	−	+	−	+	−	+
*Notoproctus oculatus* Arwidsson, 1906	+	−	+	−	+	−	−	−	−
Onuphidae g. sp.	+	−	−	−	−	−	−	−	−
*Ophelina abranchiata* Støp-Bowitz, 1948	+	+	+	+	+	+	+	+	+
*Ophelina acuminata* Örsted, 1843	−	−	−	+	−	−	−	−	−
*Ophelina cylindricaudata* (Hansen, 1879)	+	−	−	−	−	+	−	−	−
*Owenia fusiformis* Delle Chiaje, 1844	+	+	−	+	+	+	+	−	−
*Oweniidae g.* sp.	−	+	+	+	−	−	−	−	−
*Paradoneis lyra* (Southern, 1914)	+	−	−	−	+	+	−	+	+
*Paramphinome jeffreysii* (McIntosh, 1868)	+	+	+	+	+	+	+	+	+
*Petaloproctus tenius* (Théel, 1879)	−	+	−	−	−	−	−	−	−
*Pholoe assimilis* Örsted, 1845	−	−	+	+	+	+	+	+	+
*Pholoe longa* (O.F. Müller, 1776)	−	+	−	−	−	−	−	−	−
*Pholoe* sp.	+	−	−	−	−	−	−	−	−
*Phyllodoce groenlandica* Örsted, 1842	+	−	+	+	+	−	+	+	+
*Phylo norvegica* (M. Sars in G.O. Sars, 1872)	−	+	−	+	−	−	−	−	−
*Pista bansei* Saphronova, 1988	+	−	−	−	−	−	−	−	−
*Pista maculata* (Dalyell, 1853)	+	−	−	−	−	−	+	−	−
*Polycirrus medusa* Grube, 1850	+	−	−	−	−	−	−	−	−
*Polycirrus norvegicus Wollebaek*, *1912*	+	−	−	−	+	−	−	−	−
*Polydora* sp.	−	+	−	−	−	−	−	−	−
Polynoidae g. sp.	+	−	−	−	−	−	−	+	+
*Praxillella gracilis* (M. Sars, 1861)	−	+	−	−	−	+	+	+	+
*Praxillella praetermissa* (Malmgren, 1865)	+	+	−	+	−	−	−	−	−
*Praxillura longissima* Arwidsson, 1906	−	+	−	−	−	−	−	−	+
*Proclea graffii* (Langerhans, 1884)	+	−	−	−	−	−	−	−	−
*Prionospio cirrifera* Wirén, 1883	+	+	+	−	+	−	−	−	+
*Pseudoscalibregma parvum* (Hansen, 1879)	+	−	+	+	−	−	+	+	+
*Rhodine gracilior* Tauber, 1879	−	+	−	−	+	−	−	+	−
*Samytha sexcirrata* (M. Sars, 1856)	+	−	−	−	−	−	−	−	−
*Scalibregma inflatum* Rathke, 1843	+	+	+	−	+	−	+	−	+
*Scolelepis burkovskii* Sikorski, 1994	−	−	−	−	−	−	−	+	−
*Scolelepis korsuni* Sikorski, 1994	−	−	−	−	−	−	−	−	+
*Sosane wireni* (Hessle, 1917)	+	−	+	+	+	+	−	−	−
*Sphaerodoropsis philippi* (Fauvel, 1911)	−	−	−	−	−	+	+	−	+
*Spio limicola* Verrill, 1879	−	−	−	−	+	+	+	−	+
*Spiochaetopterus typicus* M Sars, 1856	−	+	−	+	+	+	+	+	+
*Spiophanes kroyeri* Grube, 1860	+	+	+	+	+	+	+	+	+
Spirorbidae g. sp.	−	−	−	−	−	+	+	−	−
Syllidae g. sp.	+	+	+	+	+	+	+	+	+
*Terebellides stroemii* Sars, 1835	+	+	+	+	+	+	+	+	+
*Terebelides gracilis* Malm, 1874	+	+	+	+	+	+	+	+	+
Terebellidae g. sp.	−	−	−	−	−	+	+	+	+
*Thelepus cincinnatus* (Fabricius, 1780)	−	−	−	−	+	−	−	−	−
*Travisia forbesii* Johnston, 1840	−	−	+	−	−	−	−	−	−
